# Male support for cervical cancer screening and treatment in rural Ghana

**DOI:** 10.1371/journal.pone.0224692

**Published:** 2019-11-18

**Authors:** Charity Binka, David Teye Doku, Samuel H. Nyarko, Kofi Awusabo-Asare

**Affiliations:** 1 School of Public Service and Governance, Ghana Institute of Management and Public Administration, Achimota, Accra, Ghana; 2 Department of Population and Health, University of Cape Coast, Cape Coast, Ghana; 3 Institute for Demographic and Socioeconomic Research, University of Texas at San Antonio, San Antonio, Texas, United States of America; USC Keck School of Medicine, Institute for Global Health, UNITED STATES

## Abstract

Men have a critical role to play in reducing cervical cancer burden. Yet, there is little information on male involvement in the cervical cancer screening and treatment process in Ghana. In this study, we explore male knowledge and support during cervical cancer screening and treatment in a rural setting in Ghana. In-depth interviews and focus group discussions were conducted among a total of 41 respondents to collect qualitative data from cervical cancer patients, their male partners and other married men in the North Tongu District, Ghana. A thematic approach was used for data analysis and presentation of the results. The results show that male partners have little or no knowledge about cervical cancer. Some men provide various forms of support–financial, social, material and emotional–to their partners during the screening and treatment stages of the disease. Some men, however, abandoned their partners during the screening and treatment process of the disease. Men whose partners did not have cervical cancer said they were willing to provide financial, social, emotional and material support to their partners if they should contract the disease. Some men said they were willing to support their female partners but lacked education on the disease. This study underscores the need for cervical cancer education programmes to target Ghanaian men. The education should focus on the causes of the disease, screening and treatment methods of the disease, and, ultimately, promote spousal support during the screening and treatment processes.

## Introduction

Cervical cancer remains the fourth most prevalent cancer among women with an estimated incidence of 570,000 in 2018 only, representing 6.6 percent of all cancers among women globally. About 90% of deaths due to cervical cancer occurred in low- and middle-income countries. It is believed that the high cervical cancer mortality rate could be reduced globally through a comprehensive approach including prevention, early diagnosis, effective screening and treatment programmes [1]. In Ghana, it is estimated that about 3,151 women are diagnosed with cervical cancer yearly out of which about 2,119 die from the disease. Cervical cancer is the second prevalent cancer but ranked first in terms of mortality among women in Ghana [2]. While there are treatment opportunities and methods such as surgery, cryotherapy, chemotherapy and radiotherapy in Ghana, the cost of treatment is generally expensive and requires long-term follow-up and contact with the cancer centres [3], which are mostly in urban centres and not easily accessible by rural dwellers. There is no national programme for cervical cancer, a disease which is also not covered by the National Health Insurance Scheme. It has been observed that men play a significant role in the healthcare decisions of women [4]. Men can also contribute to reducing cervical cancer burden by protecting their partners from HPV infections, motivating them to screen and allowing them to protect themselves against the disease [5,6]. Knowledge about cervical cancer and screening is critical to the uptake of cervical cancer screening [7].

A number of studies indicate that most men have no knowledge of cervical cancer, and some men believe that they do not contribute to cervical cancer in women [4,6]. The limited knowledge of cervical cancer among men is essentially risky for women since they have limited sexual power and are usually subservient to men [8]. This ignorance about the disease could mean that men are not likely to adjust their sexual behaviour to reduce the risk of HPV transmission to women [9].

Furthermore, men’s support for female partners during cervical cancer screening and treatment may be paramount in reducing cervical cancer deaths. Yet, evidence of this has been scarce in the literature and existing literature failed to examine specific supports provided by males for their female partners during screening and treatment of the disease. It has been found that some men are willing to support their female partners in cancer screening and treatment activities [4, 10,11]. Nevertheless, most men’s lack of knowledge about cervical cancer and their partners’ health histories has become a problem. In Ghana, also, men’s role in the health-seeking behaviours of women is very important, because male support for cervical cancer patients is crucial for their survival chances. However, the empirical literature on male support during cervical cancer screening and treatment is inadequate in Ghana. In this paper, therefore, we explore men’s knowledge about the disease and the support they have provided for their cervical cancer patient partners during the screening and treatment stages in a rural community in Ghana.

## Materials and methods

### Study population and design

This study was conducted in Battor, in the North Tongu District of the Volta Region, Ghana. The study population included cervical cancer patients and their male partners as well as married men residing in the neighbouring communities around the district capital. This is a qualitative study that is grounded in the interpretivist research paradigm. The main goal of the study design is to put the study in a social context and help to understand the findings from the subjective experiences of the respondents [12].

### Sampling procedure

Cervical cancer patients and their partners were purposively selected through phone calls using the patient records at the Gynaecology Department of the Battor Catholic Hospital. This process started with a thorough review of the register to identify patients who had been diagnosed with cervical cancer. Fifteen (15) cervical cancer patients and ten (10) partners who were willing to participate in the study out of 60 eligible potential respondents were selected from the register for the study. Furthermore, 16 married men in the Battor vicinity who were willing to participate in the study were conveniently selected for two different focus group discussions, to explore their knowledge about the disease and their willingness to support their partners if they were diagnosed with the disease.

### Ethical issues

Ethical issues were fully addressed before the commencement of the study. In this regard, institutional approval and ethical clearance for the study were obtained from the Battor Catholic Hospital and the Ethical Clearance Committee (ERC) of the Ghana Health Service (GHS) respectively. Written informed consent was sought from all respondents of the study. Respondents were assured of anonymity and confidentiality of the information and were provided the option to quit the study if they felt uncomfortable during the process.

### The data collection process

Three research assistants were recruited and trained for the study in order to facilitate the data collection process. In-depth interviews were conducted with the partners of cervical cancer patients to explore their knowledge about the disease and their support for the patients during the screening and treatment process and with the cervical cancer patients to explore the supports received from their male partners. Thus, supports were looked at from the perspective of both the patients and their male partners. The interviews were conducted at convenient places determined by the respondents. Also, two focus group discussions with each group comprising eight people were conducted among men of the same age group from two different communities in the district. The data collection instruments–in-depth interview and focus group discussion guide–share similar structure and content. They comprised three main sections with the first section focusing on the background information of the respondents while the second section examined knowledge about cervical cancer. The types of support given or received by the respondents were examined under the last section. The majority of the interviews and focus group discussions were conducted in local languages–Ewe, Twi and Dangme–and were tape-recorded for further analysis with each of them lasting between 30 and 45 minutes. Respondent recruitment and the data collection activities occurred between March 12 and June 12, 2014.

### Analytic strategy

The recorded interviews and focus group discussions were transcribed in English. The transcripts were then proofread and edited to remove typos, inconsistencies, and repetitions in the text. To ensure high-quality data, the transcripts were given to experts to review and revise the content based on the recordings. The texts were then coded using the thematic approach to qualitative data analysis [13]. The data processing was done with the R software package for Qualitative Data Analysis (RQDA). This R package is said to be useful for organising qualitative data through systematic indexing, annotation, and retrieval functions [14, 15]. The results were retrieved and presented in direct quotes based on the emerging themes from the interviews.

## Results

### Background characteristics of respondents

A summary of the background characteristics of partners of cervical cancer and other married men from the study area has been presented in Table 1. For partners of patients, seven out of the 10 respondents were between 50 and 65 years while the remaining three were aged 30 and other 49. For the other married men, 10 out of the16 respondents were aged 50–65 while the remaining six were aged 30–49. Five out of the ten partners of patients had tertiary education while only two had junior high school education. Five out of the 16 other respondents had a junior high school education while only two had senior high or primary school education. With regard to employment status, five out of the ten partners were self-employed while two were a government employee. For the other 16 married men who participated in the focus group discussions, eight were self-employed while three were unemployed. Seven of the partners of patients had between four and six children while the remaining three had between one and three children. For the other respondents, five had 4–6 children or 7 or more children while two had no children. In terms of religious affiliation, all the partners of patients were Christians while the other married men comprised 13 Christians and three traditionalists. None of the partners of patients and other married men reported being a smoker. Six out of the 10 partners reported taking alcohol while only three out of the 16 other respondents reported taking alcohol.

**Table 1:** Background characteristics of respondents.

Characteristics	Patients	Partners of patients	Other men
**Age**			
30–49	5	3	6
50–65	10	7	10
**Level of education**			
No formal education	1	-	3
Primary school	4	-	2
Junior high school	-	2	5
Senior high school	8	3	2
Tertiary	2	5	4
**Employment status**			
Government employee	-	2	5
Private employee	-	3	-
Self-employed	10	5	8
Unemployed	5	-	3
**Number of children**			
0	-	-	2
1–3	3	3	4
4–6	9	7	5
7+	3	-	5
**Religious affiliation**			
Christian Muslim	15 -	10 -	13 -
Traditional	-	-	3
**Smoking**			
Yes	1	-	-
No	14	10	16
**Alcohol**			
Yes	4	6	3
No	11	4	13

### Men’s knowledge about cervical cancer

During the interviews, partners of cervical cancer patients were asked about their knowledge of the disease before their partners were diagnosed with the disease. Men who took part in the focus group discussion were also asked their knowledge about the disease. The male partners reported that they had no knowledge concerning the causes, symptoms and risk factors of the disease prior to the diagnosis of their partners while the other married men also reported the same. This reflects in the following statements by some male partners:

“*I did not know anything about this disease*. *I did not have any idea about the cause*, *symptoms or any risk factor of the disease*. *I only overheard it on the television being debated in Parliament as to whether it should be covered in the national health insurance scheme*. *That is all I know”*. (Partner of Cervical Cancer Patient 3, 50 years).

“*In fact*, *I had no knowledge about the disease and how it is caused*, *but all I knew was that it could kill*. *And I do not even know its local name”*. (Partner of Cervical Cancer Patient 6, 58 years).

“*I have never heard of cervical cancer disease*. *Is it also a disease that affects women*? *Then women are really suffering*.*”* (Focus Group Discussion 1).

Those who got to know about the disease after the diagnosis of their partners explained that they still could not tell causes, symptoms and risk factors of the disease. Some of the respondents only got general information about the disease from the doctors. The main problem seems to come from the fact that the respondents do not know the local name of cervical cancer since there is no specific name for it. Rather, there is a general local name for all cancers–Abimakumaku–which means the sore that never heals and eventually kills the victim. This underscores the general lack of specific knowledge about cervical cancer. This can be inferred from the following statements made by some respondents:

“*I did not know its causes and symptoms*, *but I know that it is not infectious and there is a possibility of cancer spreading to other parts of the body*. *This*, *I was told by the doctor*. *But transmitting it to other people*, *I have no idea”*. (Partner of Cervical Cancer Patient 2, 58 years).

“*Well*, *about that condition*, *the doctor explained it to us*, *but I have forgotten exactly what it is*. *But I know it had to do with only women*. *Just like the men have prostate cancer”*. (Partner of Cervical Cancer Patient 4, 51 years).

### Male partners’ support for cervical cancer patients

During the interviews, partners of cervical cancer patients were asked whether they ever supported their partners and to narrate the forms of support provided. Most of them reported that they provided their partners with various forms of support during the screening and treatment process. These forms of support have been categorised into four main themes namely; financial, social, material and emotional support (Table 2).

**Table 2:** Support provided by male partners during the screening and treatment process.

Category of support	Specific supports	Frequency
Financial	Screening and treatment expenses, transportation	10
Social	Accompanying her on her hospital visits, prayers, visits	6
Services	Cooking, washing, taking care of the home, assisting in working the shop she owned	3
Emotional	Sexual abstinence, encouragement	10

The male partners explained that they supported their wives financially, socially, emotionally and materially during the screening and screening of the disease. In this regard, one male partner reported that whenever his wife was going for treatment, he gave her money for transportation and payment of bills, even though his employers eventually refunded the bills. Another male partner also reported that he always took care of the home financially and assisted the patient in managing the shop she owned. The following were some of their responses:

“*I gave her all the necessary things that she needed*. *Sometimes*, *I borrowed money; sometimes too*, *I had to sell my things*. *Luckily enough*, *her employers also came in and helped her a lot*. *But it has been five months now without working and as the head of her department*, *someone had to replace her”*. (Partner of Cervical Cancer Patient 3, 50 years).

“*I assisted her financially and when it came to cooking*, *I gave money to the kids to take care of that*. *Also*, *I had to take a casual leave to assist her in managing her shop and accompany her to the hospital for treatment”*. (Partner of Cervical Cancer Patient 6, 58 years).

Another role male partners played in supporting their female partners during the treatment phase was to abstain from all forms of sexual relations with them. A partner recounted feeling so frustrated by the condition that they had to go and seek sexual satisfaction outside the marriage. Some also reported that they found it difficult having sex with their partners after the diagnosis because of the pain and bleeding that is concomitant with the disease. Some explained that they knew that their partners could suffer post-coital bleeding even after they had commenced treatment. As a result, they tried to completely abstain from sexual intercourse with their partners in order to support the recovery process of their partners. They further explained that they had diminished sexual appetite for their partners because of the nature of the condition. Some of them said:

“*After my wife’s treatment*, *I became scared to get involved with her sexually*. *But we are okay with that*. *And when I told her that I was scared to have sex with her*, *she thought about it for some time and felt sad and then she started crying”*. (Partner of Cervical Cancer Patient 3, 50 years).

“*It had affected me a lot since any time I wanted to have sexual intercourse with my wife*, *her bleeding comes to my mind*. *I was therefore scared for her*. *So*, *I sometimes had to go out and have sex with my girlfriend”*. (Partner of Cervical Cancer Patient 6, 58 years).

“*I will say that sexual intercourse is completely out of my life for now”*. (Partner of Cervical Cancer Patient 9, 63 years).

However, not all male partners actually supported their female partners. A few cervical cancer patients recounted that they had no support of any kind from their male partners. One patient was agonised over desertion by her partner owing to the condition. This can be inferred from the following responses:

“*Because of the disease*, *my husband left me and went to marry another woman*. *So we are no more together*. *Only my child is helping me*”. (Cervical Cancer Patient 4, 44 years).

“*I have no support; I do everything on my own even though I was advised by a nurse not to lift heavy things after I was discharged from the hospital*. *I was told by other nurses that if I continue to lift heavy things*, *the stitches may tear”*. (Cervical Cancer Patient 6, 63 years).

“*Nobody provided any support for me*. *I remember there was a time I even needed 300 Ghana Cedis for my treatment*, *and nobody helped me*. *Even the church I was then attending disappointed me”*. (Cervical Cancer Patient 1, 34 years).

### The willingness of males to support partners during screening and treatment

Married men from two communities in the district were asked in a focus group discussion whether they will be willing to provide various forms of support for their partners if they were diagnosed with cervical cancer. In the discussions, most of the men expressed their willingness to totally provide different kinds of support for their partners during screening and treatment. The men, therefore, explained that they would encourage their partners to go for screening and would not hesitate to take them to the hospital for treatment. They, however, felt handicapped by their lack of knowledge about the disease. As a result, some indicated their willingness to discuss the condition with the health workers to have a better understanding of the disease and help to find a solution to it. This is indicated in the responses below:

“*Some women do not understand why they should even go for the screening even when they say it is free*. *I think that I will encourage her to go for screening if that will prevent her from contracting the disease”*. (Focus Group Discussion 1)

“*Because of the prevalence of poverty in this community*, *some women would not like to go for screening*. *I will encourage and support her to go for the screening because the disease is dangerous*”. (Focus Group Discussion 2).

According to the men, they will provide any form of support including financial support for their wives to be treated. The following were the responses of some of the respondents in the focus group discussion:

***“****I will seek medical treatment from the hospital; that will be the best thing to do*. *I will personally take her to the hospital and pay for the treatment”*. (Focus Group Discussion 1).

**“***If I am told that my wife has cervical cancer*, *definitely*, *the doctor will give her advice*. *So when she told me what the doctor told her*, *I will support her based on what the doctor told her and then I will accompany her to do whatever they told her to do”*. (Focus Group Discussion 2).

From their responses, the men seemed to recognise the serious nature of the disease and were willing to offer any kind of support being financial, social, emotional or material if their female partners were diagnosed with the disease. They appreciated the need for screening as a preventive measure and therefore ready to encourage their partners to go for screening.

## Discussion

The study sought to explore the knowledge of men about cervical cancer and the support given to cervical cancer patients by their male partners during the screening and treatment process. The findings show that men had little or no knowledge about the disease. A number of previous studies have also shown that men have low or inadequate cervical cancer knowledge [16–18]. In this study, it has been observed that the men did not have any information about the causes and symptoms of the disease as well as the risk factors of the disease. Even the few male partners who had a little knowledge about the disease had it from the doctors or health personnel after the diagnosis of their female partners.

The findings show that the information given to them by the doctors was generally about the nature of the disease and not about the specific causes, symptoms and risk factors. Hence, the respondents had no previous knowledge about the disease. This is consistent with findings of studies by Williams and Amoateng [4] and Maree et al. [6] and in which they found that men have inaccurate or no knowledge about cervical cancer. Admittedly, it can be quite difficult for the respondents to have adequate accurate knowledge about a disease that has no specific local name. The implication of this situation is that men will hardly identify what exactly is wrong with their wives or female partners to quickly take the necessary steps in order to save the lives of their female partners.

From this study, there is evidence that most male partners had supported their female partners in diverse forms during the screening and treatment process. Male partners were reported to have played the role of financiers during the screening and treatment of the disease. Both the female and male partners confirmed that the male partners provided a number of supports during the screening and treatment process. The issues of social and emotional support from the male partners also emerged in the results. Some male partners supported their female partners as caregivers during the critical stages of the disease by sharing the pain of their partners and accompanying them to the hospitals or clinics whenever necessary.

Despite this, the study also provides some evidence that a few male partners of the cervical cancer patients have abandoned their partners without any form of support during the screening and treatment process, forcing some patients to manage to support themselves in virtually everything. Sexual relationships are very important in marriage in the Ghanaian cultural context. Being a genital cancer, patients are not able to perform their conjugal duties. Furthermore, cervical patients produce awful odours in their final stage that are likely to put their male partners off. Meanwhile, traditionally, polygamous marriage is acceptable in Ghana, particularly in rural settings where there is a strong preservation of indigenous practices. Consequently, some male partners who are unable to tolerate the situation use it as an excuse to marry new wives or get new sexual partners. Some cervical cancer patients also reported that their partners left them because of the high cost of treating the condition. In contrast, some research has established the fact that male partners play a critical role in supporting their female partners in cancer screening and treatment activities [4,10]. Hence, it is crucial to recognise the significant role of men in the health behaviours of Ghanaian women. The notable implication is that including males in cervical cancer education programmes is, therefore, a crucial component in cervical cancer screening (prevention) and treatment [11].

In this study, attention was drawn to the peculiar nature of the disease as genital cancer that demands the support and understanding of partners of the patients. The findings further show that as a form of support, partners of cervical cancer patients have to accept to abstain from sexual relations with their partners. The men explain that abstaining from sexual intercourse with their partners would reduce the pain and bleeding and prevent any interruption in the treatment regimen and in turn hasten the healing process. Sexual abstinence with the patient will also help to quell the heavy blood flow and pains associated with the disease. Without an understanding of the situation, male partners may not be willing to sacrifice their sexual pleasure which could lead to possible separation, divorce or neglect of the partner. Besides, some men could also use the situation as an excuse to indulge in infidelity. Furthermore, it is noteworthy that men whose partners were not suffering from cervical cancer were also keen on offering any form of support to their partners if they contract the disease. In addition, they were also willing to encourage their partners to go for screening. Similar findings have also been established in the literature among men in Kenya [16], and Uganda [17] as well as sub-Saharan African immigrant men [18]. In this study, most of the men believe that they will quickly seek medical attention for the disease and provide any necessary support that will be prescribed by the medical personnel, in order to help treat their female partners.

## Conclusions

This study provides evidence that male partners had little or no knowledge about cervical cancer. Nevertheless, some of them supported their partners financially, socially, emotionally and materially when they contract the disease. On the contrary, some of the men abandoned their partners during the screening and treatment process of cervical cancer. Men whose partners did not have cervical cancer said they were willing to provide any financial, social, emotional and material support to their partners if they ever contract the disease. They were also ready to encourage their partners to go for screening to prevent the disease from occurring. They, however, asked for more education on the causes and treatment options. Admittedly, in this socio-cultural context where there is no specific local name for cervical cancer, the conceptions of disease and illness may be potentially confounded by many unknown factors, particularly among respondents who had little or no knowledge about the disease. Despite this, male partners of cervical cancer patients have shown in this study that they were educated on the disease by doctors after the diagnosis of their female partners and that they had some knowledge about the disease at the time of the study.

These findings underscore the critical role men can play in considerably reducing the cervical cancer incidence and mortality. This could be done through education that would equip the needed knowledge of the disease. This can contribute to the removal of the barriers to screening and treatment in, particularly, rural settings in Ghana. Men have not been adequately targeted in cervical cancer campaigns simply because it is not men’s disease. However, the findings of this study highlight the need for cervical cancer education among men in the district, particularly, those in marital union. Cervical cancer education interventions for Ghanaian men need to focus on providing information about the disease and contribute to increasing spousal support during cervical cancer screening and treatment.
